# Gender-Heterogeneous Working Groups Produce Higher Quality Science

**DOI:** 10.1371/journal.pone.0079147

**Published:** 2013-10-30

**Authors:** Lesley G. Campbell, Siya Mehtani, Mary E. Dozier, Janice Rinehart

**Affiliations:** 1 Department of Ecology and Evolutionary Biology, Rice University, Houston, United States of America; 2 Department of Chemistry and Biology, Ryerson University, Toronto, Canada; 3 National Science Foundation ADVANCE Program, Rice University, Houston, United States of America; Université de Montréal, Canada

## Abstract

Here we present the first empirical evidence to support the hypothesis that a gender-heterogeneous problem-solving team generally produced journal articles perceived to be higher quality by peers than a team comprised of highly-performing individuals of the same gender. Although women were historically underrepresented as principal investigators of working groups, their frequency as PIs at the National Center for Ecological Analysis and Synthesis is now comparable to the national frequencies in biology and they are now equally qualified, in terms of their impact on the accumulation of ecological knowledge (as measured by the h-index). While women continue to be underrepresented as working group participants, peer-reviewed publications with gender-heterogeneous authorship teams received 34% more citations than publications produced by gender-uniform authorship teams. This suggests that peers citing these publications perceive publications that also happen to have gender-heterogeneous authorship teams as higher quality than publications with gender uniform authorship teams. Promoting diversity not only promotes representation and fairness but may lead to higher quality science.

## Introduction

Equal-opportunity hiring practices have been implemented repeatedly to promote fairness and represent human diversity; but could they also lead to the production of higher quality work? This argument has been suggested repeatedly by policy makers and advocacy groups, but lacks empirical support [1]–[4]. While gender diversity is known to improve internal group processes, there is ambiguous evidence (at best) for the effect of gender diversity on group performance. Gender diverse groups tend to collaborate more effectively and exhibit higher collective intelligence [5]; and this effect is primarily explained by benefits to group processes, like better morale [6], different interpersonal styles promoting greater social sensitivity, conversational turn-taking, etc. [5], [7]–[9]. The effect of gender diversity on team performance appears more complex and context-dependent [2]–[4], [10]–[16]. Specifically, studies have often revealed no effect or a negative effect of gender diversity on team performance [2]–[4]. Otherwise, the effect of gender diversity depends upon team demography, task difficulty, etc. [2], [16]. Finally, in groups in which women have more perceived expertise than other group members, the productivity of the team might be negatively affected [17].

Recently, however, theoretical work by Hong and Page [18], [19] revealed the potential truth behind the workplace folklore that gender diversity can lead to improved productivity. Specifically, groups of problem-solvers randomly selected from a large group of intelligent operators outperformed similar groups composed of the smartest individuals selected from the same group. The failure of women to flourish in academic settings has been routinely blamed on their “different availability of aptitude at the high end”[20], [21]. Therefore, one might surmise that groups containing women might not perform as well as groups without women. However, simulations that grouped individuals with diverse problem-solving skills led to the generation of more diverse solutions, from which the best solutions were more likely to be identified than from the pool of solutions created by smarter, but more uniform groups of problem-solvers [18], [19]. Therefore, these models predict the *opposite* outcome predicted by many opponents of equal opportunity hiring practices [20]–[22], assuming gender diversity can approximate diversity in the model. Based on the results provided by the studies mentioned above [2]–[16], problem-solving diversity appears to be related to within-group gender-diversity, and therefore we would expect gender- or race-diverse groups to outperform homogeneous groups in their attempts to solve problems of import (e.g., [23]–[25]).

Ecology and environmental sciences increasingly involve collaborative research efforts [26], [27]. Collaboration can benefit academics by increasing early career prospects [28] and the citation rate of papers [29], [30]. It is still unclear whether collaboration affects the recognition of a paper’s academic importance. For instance, some studies have suggested that women’s articles might be cited more than their male colleagues in certain fields [31], [32]. However, a more recent study of geography journal articles found that single, male authored publications tend to be cited more frequently than papers produced by collaborative male groups, gender diverse collaborative groups, or all female (single- or multi-authored) author groups [26]. In other studies, results suggest that gender doesn’t affect citation rate [33], [34]. And finally, some studies suggest that women are less frequently cited than their male colleagues[35].

Here, we evaluated the quantity of gender diversity at the level of leadership (PIs) and working group (WG) participants within one of the most influential ecological institutions worldwide, the *National Center for Ecological Analysis and Synthesis* (NCEAS). Because women are relatively common but not ubiquitous in Ecological Science [36], it is a particularly suitable scientific sub-discipline in which to explore the consequences of gender diversity for research productivity. Therefore, we also explored the relationship between gender diversity in these working groups and the perceived quality of science (as measured by citations) these groups produce.

## Methods

### National Center for Ecological Analysis and Synthesis

Since 1995, NCEAS has served as one of the most influential ecological institutions worldwide by promoting discussions among ecologists of diverse interests as well as the synthesis of ecological data and theory [37], [38]. Annually, NCEAS sponsors WG meetings, involving 4–40 experts, on a variety of ecological topics. These experts include PIs (typically groups of 1 – 5 people) who invite WG participants after the PI’s proposal has been funded. By collaborating, WG participants also often act as authors of WG publications. By 2007, 157 WGs (not including sabbatical or post-doctoral scholar visits) had completed their tenure at NCEAS, generating almost 2000 publications. To promote institutional transparency, NCEAS posts complete records of WG activities (e.g., participant identities, citations of products) on their website (http://www.nceas.ucsb.edu/projects), providing a rich dataset with which to explore repeated events over the last decade of male and female academic ecologists collaborating in science. By analyzing the dynamics of these formally-constructed working groups, we hope to gain an improved understanding of scientific collaborations that occur daily within universities and government laboratories, and to explore how women participate in the practice of collaborative science.

### Data Collection

For each WG, we recorded the number and identity of PIs, participants, and authors from NCEAS’ website (accessed between November 1, 2008 and February 28, 2009). Because this research involved humans, we received permission to proceed with Research on Human subjects from the Rice University Institutional Review Board (IRB) for the Protection of Human Subjects, who also waived the need for written informed consent from participants. First, we determined the gender of NCEAS PIs, WG participants, and authors using gender-obvious names (in the English language) or photographs of individuals with gender-neutral names. For all foreign names, we searched for photographs of individuals because we assumed we did not understand whether names were gender neutral or not. We found the photographs by searching the Internet with the person’s name and institution. To compare the frequency of women acting as PIs or WG participants relative to their availability within the general population of US academics, we used data collected by NSF on the frequency of male and female doctorate holders employed by universities and four-year colleges in 1997 and 2006 [39], [40].

Next, we assessed past academic contributions of PIs, using h-indexes for the preceding decade. Given that we were comparing authors’ academic contributions from a single sub-field of science, we chose to use the h-index as a measure of academic output of scientists [41]. The h-index is a simple and effective index that has been used widely and incorporates not only an author’s productivity but also the relative impact of papers published by that author. To estimate a PI’s h-index before participating in NCEAS, we recorded the h-index for the decade preceding project initiation as reported by ISI’s Web of Science (http://apps.isiknowledge.com, Feb. 2009) for publications by that author with a distinctly ecological theme (based on an internally agreed upon list of themes the authors agreed were ecological in nature). Although this may underestimate an academic’s overall contribution to general scientific knowledge, it provides an assessment of that individual’s contribution to the particular field of ecology. We distinguished journals that published ecological versus non-ecological studies based on the statement of purpose published by each journal. This may overestimate the contribution of the few ecologists who share initials and last names with other ecologists; however, we were unfortunately unable to consistently differentiate among these individuals. The h-index then was recalculated on the basis of the citation counts of these individual articles. For each peer-reviewed publication, we recorded the gender of authors (as above) and the number of citations using Google Scholar (scholar.google.ca, August 2012). Gender-heterogeneous groups were categorized as groups with at least one male and one female working group participant.

### Data Analysis

Over the lifetime of the collaborative WGs (from first meeting to last reported publication), we evaluated the changes in women’s participation across the first decade of NCEAS’s existence. We asked if, over time, the increased frequency of women (acting as working group participants and principal investigators, PIs) coincided with their past academic contributions (pre-project h-index) and the number of citations received by published articles arising from the working groups. If increases in the frequency of women (as PIs or working group participants) are due to institutional quotas alone, then the quality of women’s contributions to academic ecology should not change over time. However, if NCEAS is recruiting more women because of their expertise in the field, then we would expect an increase in the perceived quality of women’s contributions to academic ecology (measured by the number of citations received by a journal article and h-index, as well as women’s relative authorship rank).

To compare the frequency of female PIs to female academics at US universities across a decade, we used chi-square analysis to compare NSF frequency data from 1997 and 2006. The relative frequency of female PIs was estimated using data from the first three years of NCEAS’s history, from 1996–1998, and then again from the last three years of our data collection period, from 2005–2007. We then compared the h-index of male and female PIs using a multivariate analysis of variance (MANOVA), accounting for the time since the project started and the year of the PI’s first publication.

To compare the frequency of female WG participants to female academics at US universities across a decade, we again performed chi-square analysis with data similar to that mentioned above. The relative frequency of female WG participants was estimated using data from the first three years of NCEAS’s history, from 1996–1998, and then again from the last three years of our data collection, from 2005–2007. To understand how gender diversity may relate to the number of citations a paper receives, we performed an analysis of covariance (ANCOVA) where number of citations (natural log transformed +1) was the response variable, and explored the relationship of number of citations (natural log transformed +1) with gender diversity (present, absent), and the presence/absence of women in authorship positions of status (first author, last author or both) and proportion of authors who were female (arc sine square root transformed) while accounting for the effect of journal impact factor (natural log +1 transformed) and years since publication. Unfortunately, we were unable to perform additional statistical analyses on female only authorship groups, because, over the 2005–2007 time period, we were able to identify only two papers published by female-only authorship groups.

To compare the relative rank of female authors for WGs early on in NCEAS history versus later, we ran a two-sample Kolmogorov-Smirnov test with the grouping variable being the temporal group (years 1995–1997 and 2005–2007) and the response variables being average relative rank of female authors, frequency of female first-authors per paper, and frequency of female last-authors per paper. We used nonparametric analysis because the data were not normally distributed, and transformations did not improve the normality. All statistical analyses were run using SPSS v. 17.0.2.

## Results

**Figure 1:**
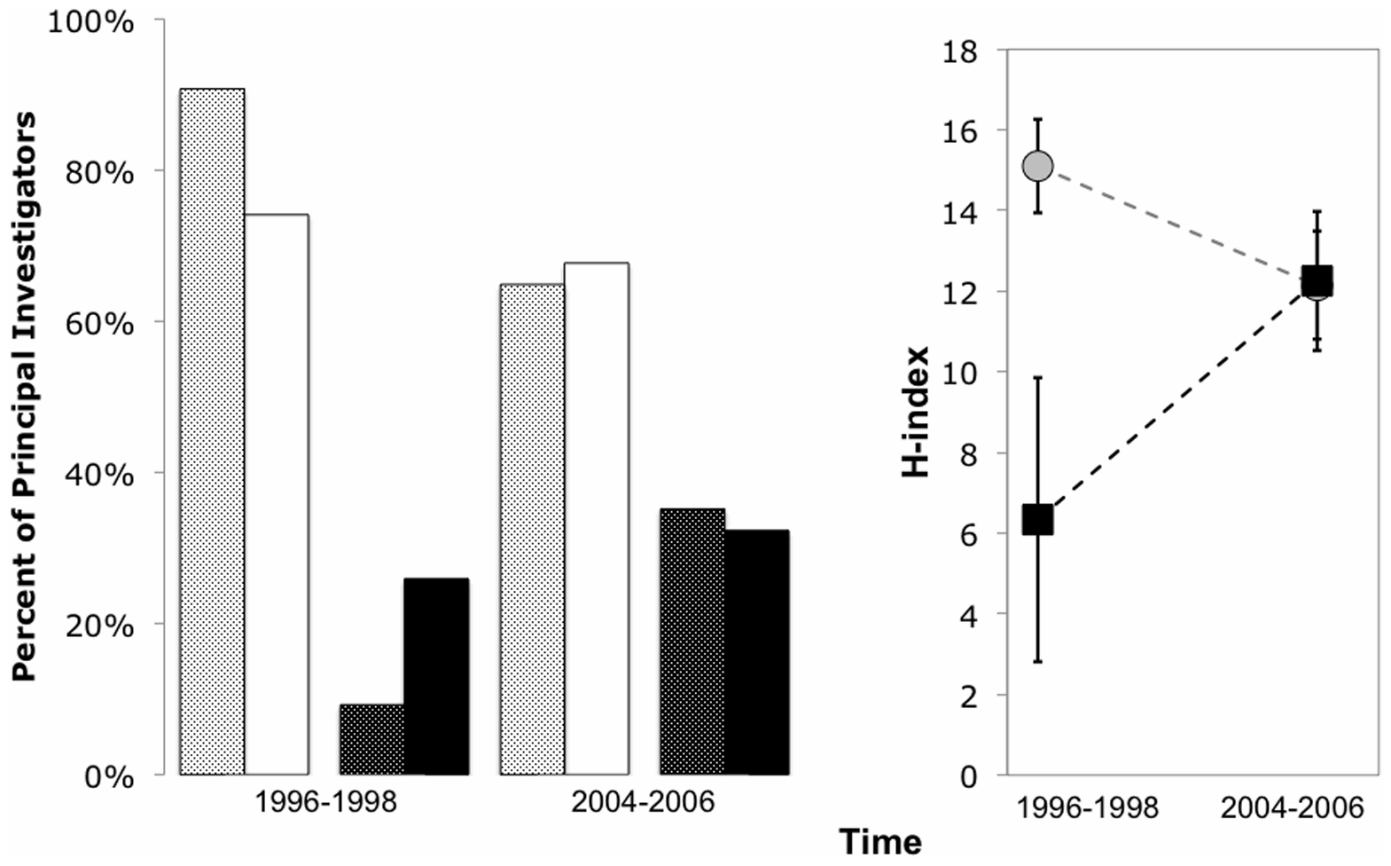
Temporal changes in A) the frequency of male and female principal investigators at the National Center for Ecological Analysis and Synthesis (men: white background, black dots; women: black background, white dots) and at US universities (men: white unpatterned; women:black unpatterned, data from NSF 1998, 2009) and B) their average (SE) h-index (men: grey circle; women: black square).

First, we compared the recent h-indexes (2005–2007) of male and female NCEAS PIs with those of the preceding decade (1996–1998). For the first three years of NCEAS, the proportion of female PIs was significantly lower than the proportion of female academics employed by US universities (χ^2^ = 25.64, *P*<0.001, Figure 1A). Further, the h-indexes of these early female PIs were approximately half that of their male counterparts (Figure 1B). Over time, the proportion of female PIs at NCEAS increased such that, recently, the proportion of female PIs was virtually identical to the proportion of female academics employed by US universities (χ^2^ =  0.0008, *P*>0.05). On average, h-indexes evaluated more recently should be lower than h-indexes evaluated a decade ago because younger scientists have had less time to publish articles and accumulate citations, as observed with male PI h-indexes (MANOVA: Gender x Time: F_1,123_ = 4.22, *P* = 0.042). In contrast, the h-index of female PIs increased to equal that of their male counterparts, coincident with their increased prevalence as PIs. Therefore, as gender parity was reached in leadership roles, we observed an increase in the participation rate of highly (and equally) qualified women.

**Figure 2:**
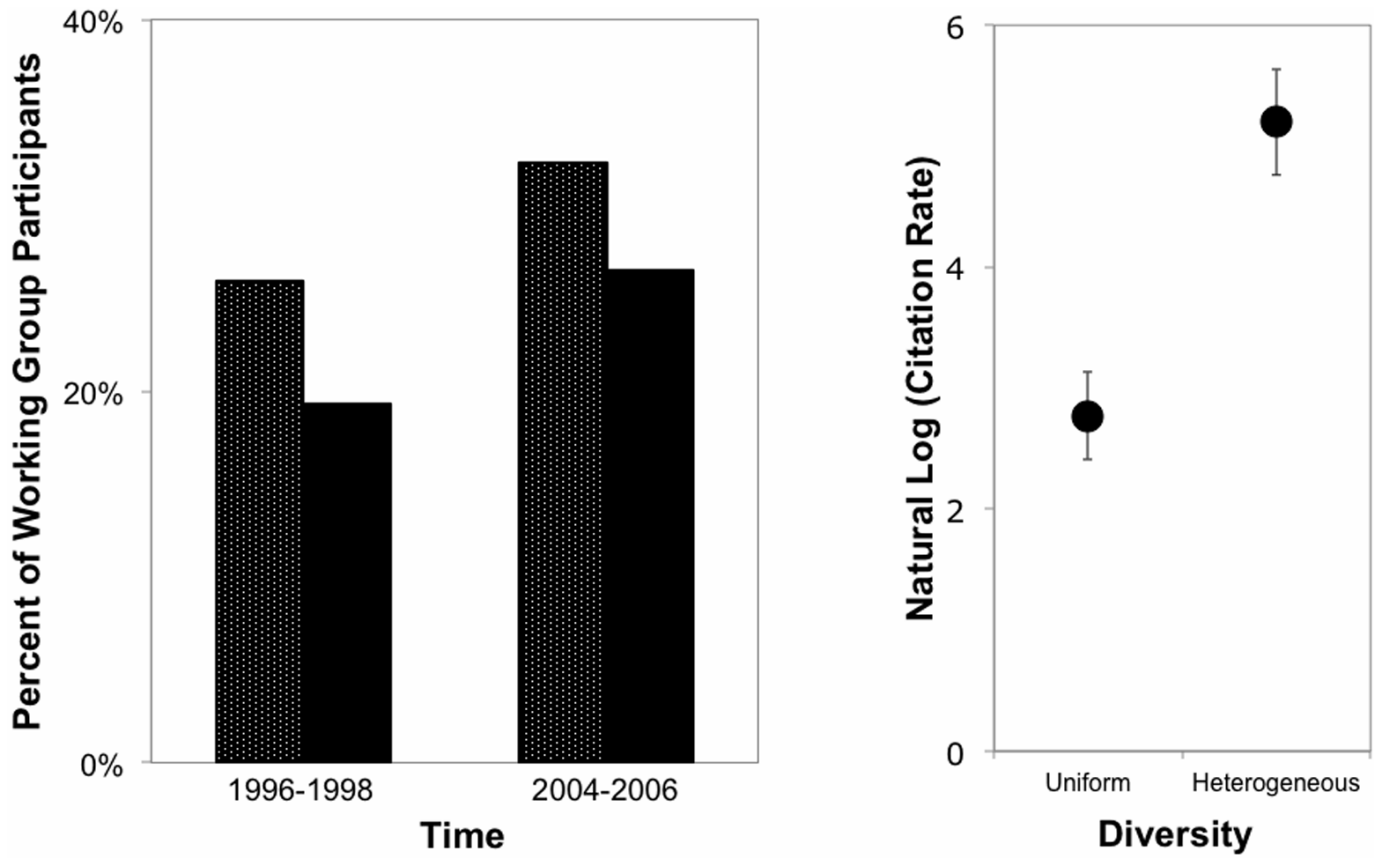
Differences in A) the frequency of female working group participants at the National Center for Ecological Analysis and Synthesis (black background, white dots) and in US universities (black, unpatterned, data from NSF 1998, 2009) over time and B) the average number of citations (SE) received by publications with or without female coauthors.

Second, we evaluated changes in the proportion of women WG participants through time and the number of citations received by publications relative to the proportion of authors who were female, with or without women in authorship positions of status (i.e., first or last author). Between 1996 and 1998, the proportion of female WG participants was significantly less than the national proportion of female faculty at US universities (χ^2^ = 123.48, *P*<0.001, Figure 2A). This low proportion of female participants rose slightly but continued to be significantly less than the national proportion of female faculty between 2005 and 2007 (χ^2^ = 35.02, *P*<0.001). We then compared the average number of citations received by publications with and without female coauthors, controlling for journal impact factor (F_1,200_ = 32.377, *P*<0.0001); Figure 2B). Despite the discrepancy between the proportion of WG participants and the availability of women to participate, publications with at least one female coauthor tended to be cited 87% more often than publications without female coauthors (F_1,200_ = 14.86, *P*<0.001). Interestingly, as the proportion of authors that were female increases, the number of citations received decreases (β = –3.46, F_1,200_ = 18.86, *P*<0.001). This particular result may reinforce the concept that the presence of diversity, rather than the presence of one gender or the other has a significant effect on group function and effectiveness. Publications with women in both first and last author positions tended to receive marginally significantly more citations than publications without women in authorship positions of status or in only one position (either first or last position; F_2,200_ = 2.31, *P* = 0.10). Finally, time period (and its interaction with either the presence/absence of diversity or the presence of women in authorship positions of status) did not significantly affect citation rate (Time period: F_1,200_  =  0.12, *P* = 0.7; Time period x Diversity: F_1,200_  =  0.6, *P* = 0.5; Time period x Status: F_2,200_ = 0.2, *P* = 0.8). Therefore, though women in non-leadership positions continue to be under-represented as participants, the perspectives provided by both genders within a working group appear to play a fundamental role as authors in increasing the quality of publications produced.

Finally we explored the proportion and visibility of women as authors relative to their male colleagues. Over our observation period, the proportion of publications with at least one female author increased significantly over time from 42.8% (SE = 5.8) to 76.2% (SE = 7.2, F_1, 48_ = 12.489, P = 0.001). Furthermore, the proportion of female authors has increased significantly from 40.6% (SE = 5.9) to 44.3% (SE = 7.6) over that same time period (F_1, 48_ = 5.364, P =  0.025). However, there were no significant changes through time in the proportion of women acting as first authors (Early: mean  =  22.4%, SE  =  0.05; Late: mean  =  20.0%, SE  =  0.07; Kolmogorov-Smirnov test: Z = 1.155, df = 60, *P* = 0.139) or last authors (Early: mean  =  18.5%, SE  =  0.04; Late: mean  =  22.5%, SE  =  0.05; Kolmogorov-Smirnov test: Z = 0.695, df = 60, *P* = 0.720). Finally, there were no significant changes to the average relative rank of female authors over time (Kolmogorov-Smirnov test: Z = 0.981, df = 60, *P* = 0.291). Even though the proportion of female authors increased coincidently with the increase in female PIs, the proportion of women occupying more prestigious authorship roles did not change.

## Discussion

Our study also revealed that as the proportion of women in leadership positions increased, the quality of women as experienced scientists filling those positions also increased to equal that of their male counterparts, resulting in an overall increase in average leadership quality (measured by a change in h-index). This result is consistent with a recent congressional report measuring the productivity of women at US universities [42]. As leadership gender-diversity increases, as it did in our study, we expect that this may create a more welcoming social environment that, in turn, might have strong influences on the retention of women in science [43], [44]. Despite this hopeful trend, women continue to be minorities in faculty positions and leadership roles in academic science [45]. Perhaps more puzzling is the continued low proportion of who women participate in working groups, given that participants often include graduate student and post-doctoral populations - both of which include high proportions of women (although there may be other reasons for this pattern [46]–[49]). Differences in the increased proportion of women PIs versus WG participants through time may be partially a consequence of differences in their relative experience in scientific collaboration and knowledge of their ecological field. However, it is also possible that institutional efforts to increase gender diversity are more focused on highly-visible leadership positions, such as PIs, than working group participant populations, and that bias against including women as WG participants still exists [44], [50]. Promoting women, not only as PIs, but also as participants and coauthors in prestigious collaborative groups like those hosted by NCEAS, is likely to substantially alter the trajectory of their careers [44], [51].

Our study is not without limitations. We confined our analysis to a ten-year period of NCEAS’ history. This may be insufficient to fully explore the nature of publication productivity in the ecological sciences, but it does provide a benchmark for consequent studies. Second, we determined gender by name or picture recognition; some people, including academics, project gender ambiguity making our method susceptible to mistakes. Further our dataset included at least one male-to-female transgendered scientist whose publication rate was higher than the average publication rates of the other female scientists in our dataset (but was not an outlier to the dataset). This may point to interesting research questions into the effect of early-gendered socialization on academic success in the scientific community. In addition, we compared the frequency of female PIs and working group members to the proportion of female academics who identify themselves as working in the Biological Sciences across all departments at U.S. institutions because this was the finest resolution provided by NSF. Given that there may be differences in the proportion of women in ecology versus cellular biology, female ecology academics may be under- or over-represented in the NCEAS working groups. Finally, we used two indices to measure scientific output. The h-index is an imperfect measure of a scientist’s productivity for a variety of reasons (including gender and age bias, [52]–[56]) and yet it is the most frequently used (with wide general acceptance) numerical indicator that provides a reasonable method for ranking scientific productivity[53].

Few female authors participate in prestigious authorship roles, even though their frequency as authors has increased over time. This dataset is the first to document the positive consequences of gender diversity on the quality of science produced by collaborative working groups. Gender-diverse groups (specifically authorship groups with at least one woman) tend to receive more citations from their peers, suggesting that peers perceive the publications produced by gender-diverse groups to be higher quality. Bringing together the collective abilities of diverse thinkers need not be thought of as an exercise in tokenism but rather as the best opportunity to address the biggest scientific puzzles of the day.
